# A Case of Anti-Synthetase Syndrome With Anti-Glycyl tRNA Synthetases Antibody Developed After COVID-19

**DOI:** 10.7759/cureus.58004

**Published:** 2024-04-10

**Authors:** Yusuke Irie, Hiroki Wakabayashi, Yasuo Matuzawa, Nobuyuki Hiruta, Kaichi Kaneko

**Affiliations:** 1 Respiratory Medicine, Toho University Sakura Medical Center, Sakura-shi, JPN; 2 Pathology and Laboratory Medicine, Toho University Sakura Medical Center, Sakura-shi, JPN; 3 Rheumatology, Toho University Sakura Medical Center, Sakura-shi, JPN

**Keywords:** long covid, aminoacyl-trna synthetases, long-term covid-19, covid-19, anti-ej antibody, anti-synthetase syndrome

## Abstract

Coronavirus disease 2019 (COVID-19) is a life-threatening respiratory disease characterized by severe acute infection. In some cases, COVID-19 symptoms may persist for a long term, posing a significant social problem. Long-term COVID-19 symptoms resemble those observed in various autoimmune diseases, such as dermatomyositis and polymyositis. In this report, we present the case of a 55-year-old woman who had been experiencing persistent dyspnea on exertion since contracting COVID-19 a month ago and was subsequently diagnosed with anti-synthetase syndrome (ASS). The patient presented with fever, dyspnea, rash, mechanic’s hands, and arthritis. Computed tomography imaging revealed findings indicative of interstitial pneumonia. Immunological test results were positive for anti-EJ antibody, leading to a diagnosis of ASS based on Solomon’s established criteria. The patient’s condition improved following treatment with prednisolone, tacrolimus, and intravenous cyclophosphamide. Pathological findings of transbronchial biopsy revealed nonspecific interstitial pneumonia with organizing pneumonia, leading to speculation that ASS had developed after COVID-19. Given the scarcity of reports on ASS development post COVID-19, we conducted a literature review and compared our present case to previous ones. This report highlights the importance of considering ASS in the differential diagnosis of patients with long-term COVID-19 symptoms.

## Introduction

Coronavirus disease 2019 (COVID-19), caused by severe acute respiratory syndrome coronavirus 2 (SARS-CoV-2), is a life-threatening respiratory illness [1]. Beyond its severity as an acute infection, COVID-19 has emerged as a significant social issue, resulting in long-term effects in non-hospitalized individuals (10-30%), hospitalized patients (50-70%), and even vaccinated cases (10-12%) [2]. Long COVID is a multisystemic condition comprising severe symptoms that often follow a SARS-CoV-2 infection. Symptoms of Long COVID include fever, dyspnea, weakness, joint pain, and rashes, resembling those of autoimmune diseases. In some cases, patients have developed autoimmune diseases, such as dermatomyositis and polymyositis, following COVID-19 [1]. Therefore, distinguishing between Long COVID and dermatomyositis and polymyositis can be challenging.

Anti-synthetase syndrome (ASS) is an autoimmune disease characterized by autoantibodies against one of several aminoacyl-tRNA synthetases (ARS), with clinical features such as interstitial lung disease (ILD), non-erosive arthritis, myositis, Raynaud’s phenomenon, unexplained fever, and mechanic’s hands [3]. ASS is characterized by the presence of anti-ARS autoantibodies, which are classified into eight subgroups: antihistidyl tRNA synthetase (Jo-1), threonyl tRNA synthetase (PL-7), alanyl tRNA synthetase (PL-12), anti-glycyl tRNA synthetase (EJ), isoleucyl tRNA synthetase (OJ), asparaginyl tRNA synthetase (KS), phenylalanyl tRNA synthetase (ZO), and tyrosyl tRNA synthetase (HA). Each subgroup has a different reported positivity rate for autoantibodies: JO-1 (15-30%), PL-7 (2-5%), PL-12 (2-5%), EJ (2-5%), OJ (<2%), KS (<2%), HA (<1%), and Zo (<1%).

Currently, only a few cases of ASS following COVID-19 have been reported [4-7]. Here, we present a case of ASS with confirmed positive anti-EJ autoantibodies that developed after COVID-19. Additionally, we review the existing literature on ASS cases associated with COVID-19.

## Case presentation

A 55-year-old woman, with no relevant medical history, received three doses of an mRNA vaccine against SARS-CoV-2. One month before hospital presentation, she contracted COVID-19 and was advised for observation by her previous doctor. She did not receive any treatment including glucocorticoid or remdesivir. However, she had persistent dyspnea on exertion leading up to her hospital presentation. One week before being admitted, the patient developed symptoms including fever ranging from 38.0°C to 38.9°C, dyspnea, and a swelling-like skin rash with itching on her face, trunk, and extremities. She tested positive for serum anti-ARS autoantibodies. However, the skin rash was not unique to dermatomyositis such as heliotrope rash, Gottron's sign, or V-neck sign.

**Figure 1:**
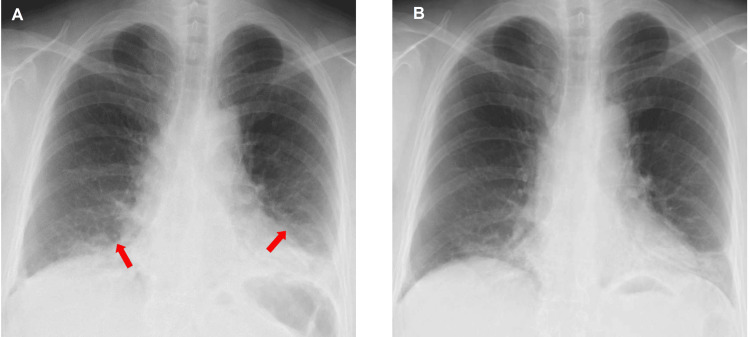
Chest radiography of the patient before and after treatment (A) Chest radiography at the first visit: attenuation of the inferior lung observed; (B) Chest radiograph on the day of discharge: improved attenuation of the inferior lung.

**Figure 2:**
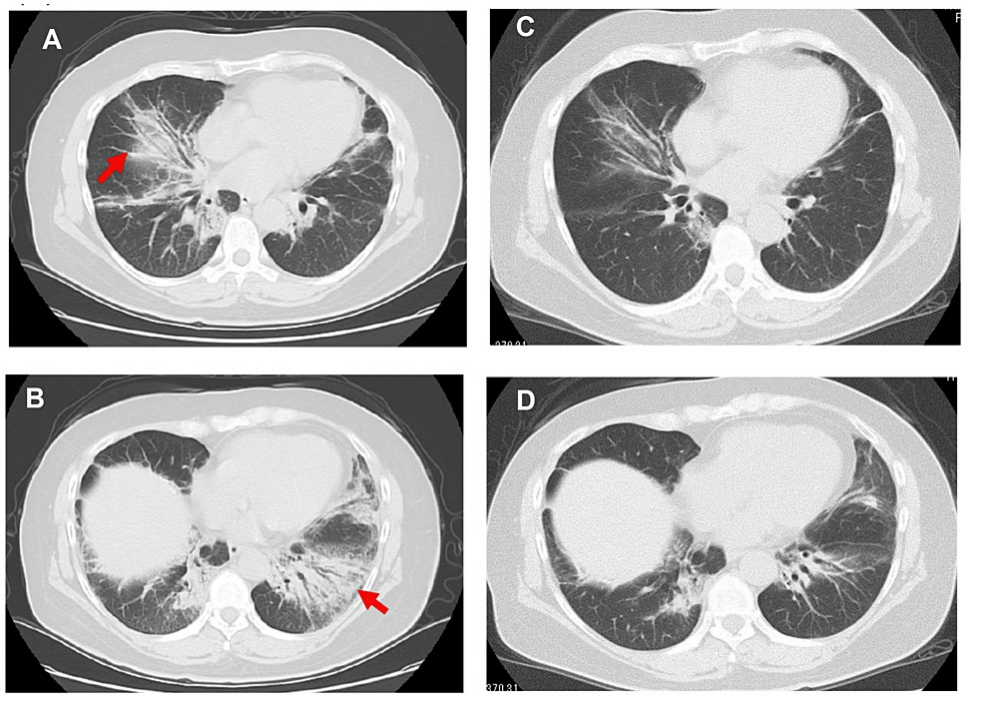
Chest CT of the patient before and after treatment. (A and B) Chest CT at the first visit: peribronchial infiltrative shadows are predominant in the bilateral lung bases; (C and D) Chest CT on day 19 of hospitalization: peribronchial infiltrative shadows are improved.

During the initial examination, her body temperature was 38.3°C, with a percutaneous oxygen saturation of 94% at room air. Auscultation confirmed fine crackles predominantly in the dorsal lower lung field. Mechanic's hand was observed on the right index finger. Laboratory investigations demonstrated elevated C-reactive protein (6.6 mg/L) and Krebs von den lungen-6 (798 U/mL) levels, with normal creatine kinase (CK) and aldolase levels. Immunology testing using the EUROLINE immunoblot assay (EUROIMMUN Medizinische Labordiagnostika AG, Lübeck, Germany) revealed the presence of anti-nuclear, anti-ARS, and anti-EJ antibodies, whereas anti-melanoma dedifferentiation-associated gene 5, anti-Mi-2, and anti-transcription intermediary factor 1-γ antibodies were absent. Chest radiography revealed attenuation of the inferior lung (Figure 1A), and computed tomography (CT) demonstrated consolidation and bronchovascular bundle thickening in the bilateral lower lobes (Figures 2A, 2B). There were no apparent solid tumor observed in the systemic CT scan.

**Figure 3:**
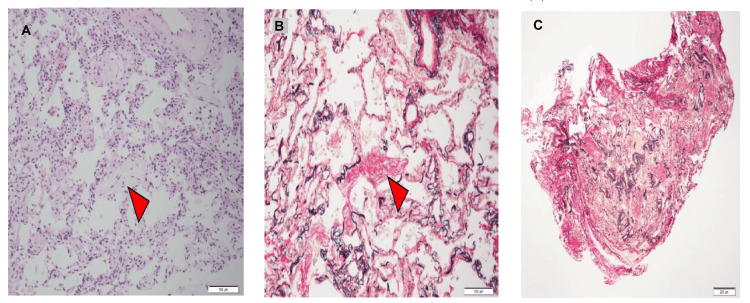
Pathological transbronchial lung biopsy findings. Pathological examination revealed mild lymphocytic infiltration, diffuse collagen fibrosis in the alveolar septum, and granulation tissue formation in the airspace, indicated by the arrow, consistent with nonspecific interstitial pneumonia with organizing pneumonia. (A) Hematoxylin and eosin staining, 100× magnification; (B) Elastica van Gieson staining, 100× magnification; (C) Elastica van Gieson staining, 40× magnification.

A bronchoscopy was performed on day 1 to investigate the cause of ILD. Analysis of bronchoscopic alveolar lavage revealed a lymphocyte-predominant cellular fraction with negative bacterial culture. Transbronchial biopsy findings indicated mild lymphocytic infiltration, diffuse collagen fibrosis in the alveolar septum, and granulation in the airspace, suggesting nonspecific interstitial pneumonia (NSIP) with organizing pneumonia (OP) (Figure 3). Moreover, skin biopsy revealed perivascular lymphocytic infiltration and a red thrombus without vasculitis or granulomas. The patient was positive for anti-EJ antibody, thus meeting the criteria for ASS criteria as established by Solomon, including anti-EJ antibody positivity, ILD, joint pain, and skin rash [8]. However, the diagnostic criteria for dermatomyositis by the American College of Rheumatology (ACR)/European League Against Rheumatism (EULAR) were not met [9].

**Figure 4:**
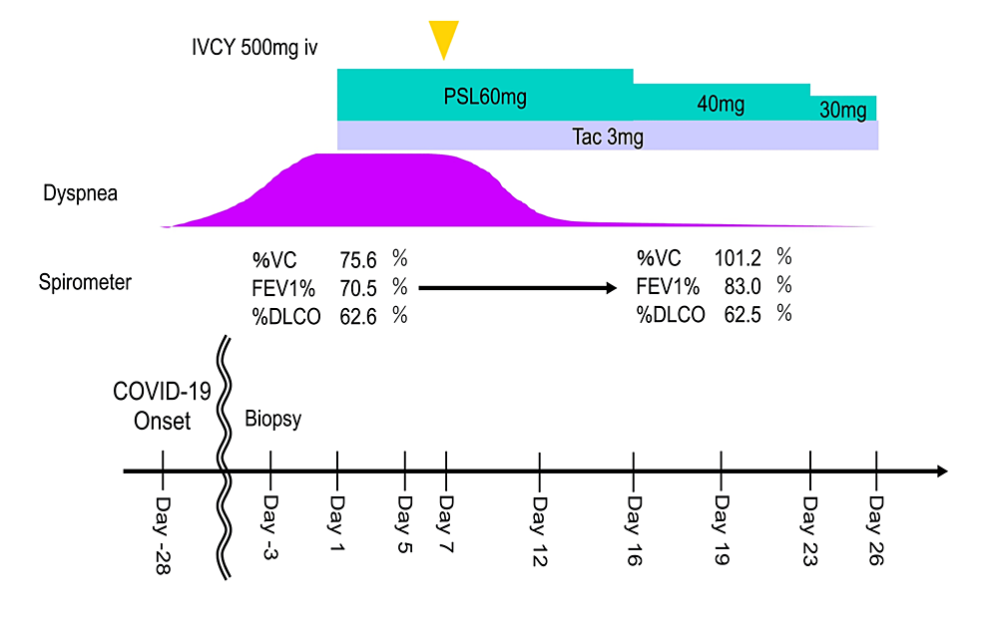
Clinical course of the patient. IVCY, intravenous cyclophosphamide; PSL, prednisolone; Tac, tacrolimus; %VC, % vital capacity; FEV1%, forced expiratory volume in one second %, %DLco, diffusing capacity of the lung for carbon monoxide; COVID-19, coronavirus disease 2019

The patient was initiated on prednisolone (60 mg/day) and tacrolimus (Tac, 3 mg/day) on day 1 (Figure 4). However, by day 6, no improvement in dyspnea or desaturation on exertion was observed; therefore, intravenous cyclophosphamide (IVCY) was administered. By day 12, both the chest radiography findings and the patient’s symptoms had improved, promoting a tapering of prednisolone. By day 19, the serum Krebs von den lungen-6 levels had decreased from 798 to 566 U/mL. Peribronchial infiltrative shadows on chest CT were improved, and respiratory function test results showed improvements in both vital capacity and forced expiratory volume in one second (FEV1%) (Figure 2C, 2D).

## Discussion

In this report, we present the case of a patient with anti-EJ antibody-positive ASS that developed following contracting COVID-19. Thus far, several cases of ASS following COVID-19 have been reported (Table 1) [1,4-7,10]. Notably, these previously reported cases involved young male patients only. Moreover, despite some variability across the cases, the time from the COVID-19 diagnosis to the ASS diagnosis was approximately 28 days. The clinical findings in these cases were arthritis (three patients), skin rash (two patients), elevated CK levels (six patients), and autoantibodies against Jo-1 (three patients), PL-7 (two patients), and EJ (one patient). Elevated CK levels were observed in all previously reported patients, suggesting that these cases may have been complicated by myositis.

**Table 1:** Clinical characteristics of six cases of anti-synthetase syndrome following COVID-2019. COVID-19, coronavirus disease 2019; ASS, Anti-synthetase syndrome; PL-7, threonyl tRNA synthetase; Jo-1, anti-histidyl tRNA synthetase; EJ, anti-glycyl tRNA synthetase; PL-12, alanyl tRNA synthetase; NSIP, nonspecific interstitial pneumonia; OP, organizing pneumonia; PSL, prednisolone; Tac, tacrolimus; IVCY, intravenous cyclophosphamide.

	Shimizu et al., 2022 [1]	Bouchard et al., [4]	Peña et al., 2023 [10]	Elsayed et al., 2023 [5]	Tranah et al., 2023 [6]	Blake et al., 2021 [7]	Present case
Age/Sex	47/M	62/M	36/M	32/M	38/M	72/M	56/F
Duration from infection to diagnosis	28 days	56 days	56 days	28 days	10 days	21 days	28 days
Arthritis	(-)	(+)	(-)	(+)	(-)	(+)	(+)
Rash	Heliotrope rash, V-neck rash, mechanic's hands, whiplash-like rash on back	(-)	Mechanic's hands	(-)	(-)	(-)	Swelling-like skin rash with itching on face, trunk, extremities, mechanic's hands
Autoantibody	Anti-PL-7	Anti-Jo-1	Anti-EJ	Anti-Jo-1	Anti-Jo-1, Ro-52,Ku, PL-12	Anti-PL-7	Anti-EJ
CK elevation	(+)	(+)	(+)	(+)	(+)	(+)	(-)
Chest CT Pattern	NSIP	OP	NSIP+OP	OP	OP	NSIP	NSIP+OP
Pathology	Muscle fiber atrophy around fascia, infiltration of CD8-positive lymphocytes	Perifascial necrosis, nuclear actin aggregation, MHC class I/II expression	Perivascular/intramuscular lymphocytic infiltration, muscle fiber necrosis, perifascial atrophy	(-)	(-)	(-)	Perivascular lymphocytic infiltrate, red thrombus
Treatment	PSL 60mg+Tac 3mg, IVIG	PSL 75mg+CYC, IVIG	PSL 40mg	Dexamethasone, antibacterial drug	mPSL 500mg, CYC	PSL 30mg+CYC	PSL 60mg+Tac 3mg, IVCY
Outcome	effective	effective	effective	effective	effective	effective	effective

In the present report, the patient’s clinical presentation included arthritis and skin disorders, with no elevation in CK levels. Many studies have reported that there are no differences in frequency of ASS based on sex [11]. The absence of previous reports documenting female patients with anti-EJ antibody-positive ASS following COVID-19 onset underscores the rarity of the present case. Most of the previously reported cases were treated with an effective combination of glucocorticoids and immunosuppressive drugs, a treatment approach also employed in managing our patient.

The CT findings of acute COVID-19 often reveal ground-glass opacities at the peripheral areas of the lung. In the present case, the predominant peribronchial infiltrative shadows in the bilateral lung bases differ from the typical findings of acute COVID-19 but are common in patients with ASS compared with COVID-19. We considered these CT findings in the present case to be associated with ASS rather than acute COVID-19. However, as reported by Myall et al., the majority of persistent CT findings post-COVID-19 are consistent with OP [12], making it challenging to distinguish them from ASS based on CT findings alone. Therefore, comparison using pathological findings such as TBLB is crucial to differentiate ASS from COVID-19.

In a previously reported case of COVID-19, the pathological findings of lung tissue included features of diffuse alveolar damage, such as hyaline membrane formation, fibrin exudates, epithelial damage, and diffuse type II pneumocyte hyperplasia [13]. Additionally, the pathological findings of COVID-19 comprise inflammation and disappearance of organization, fibrosis, and alveolar collapsibility as the predominant symptoms [14,15]. In the present case report, consideration was given to whether alveolar collapse in the patient was associated with inflammatory changes following COVID-19. However, no ASS-specific pathological evidence has been reported in previous cases. Recent reports have described histopathological findings of lung tissue in patients with ASS-ILD as NSIP, OP, usual interstitial pneumonia, acute lung injury, and diffuse alveolar damage [16], with NSIP being the most common pathology. The presence of OP-like and NSIP findings in transbronchial lung biopsy (TBLB) indicates acute or active disease, resembling the significant findings in ASS. Therefore, these findings suggest that ASS is likely to develop after COVID-19. However, the development of dermatomyositis after COVID-19 remains controversial [17].

Several reports have described the mechanism of autoimmune diseases that develop after COVID-19, involving molecular mimicry and cytokine-associated disruption of self-tolerance [1,18]. COVID-19 induces the activation of intracellular signaling pathways and production of inflammatory cytokines, such as tumor necrosis factor-α, interferon-β, and interleukin-6, which may trigger autoimmunity via systemic inflammation [18]. Additionally, the reduction in T-regs reduces self-tolerance, and the molecular mimicry between viral antigens and damaged muscle antigens by COVID-19 may lead to the production of autoantibodies, contributing to the development of novel autoimmunity [1]. A previous report has linked the presence of new and increased autoantibodies with disease severity, advanced age, and female sex, supporting the possibility that responses to the extracellular proteome could lead to the development of new autoantibodies [19]. However, despite this report, the mechanism underlying the production of autoantibodies after COVID-19 remains controversial.

## Conclusions

This report describes a patient with anti-EJ antibody-positive ASS that developed after contracting COVID-19. The patient exhibited clinical improvement following administration of glucocorticoids, tacrolimus, and IVCY. The clinical course and biopsy findings strongly suggest that ASS can develop after COVID-19. A comprehensive analysis of a larger number of cases will contribute to a clearer understanding of the relationship between ASS and COVID-19, helping to uncover potential shared mechanisms or triggers.
